# Possible Crosstalk Between Small Intestinal Bacterial Overgrowth (SIBO) and Atopic Manifestations—A Short Overview

**DOI:** 10.3390/ijms27041865

**Published:** 2026-02-15

**Authors:** Michał Terlecki, Wiktoria Brzeczek, Martyna Kowalczyk, Emilia Kiełczyńska, Klaudia Kukla, Gabriela Osmulska, Krzysztof Gomułka

**Affiliations:** 1 Student Research Group of Allergology and Internal Medicine, Wroclaw Medical University, 50-556 Wroclaw, Poland; michal.terlecki@student.umw.edu.pl (M.T.); wiktoria.brzeczek@student.umw.edu.pl (W.B.); martyna.kowalczyk@student.umw.edu.pl (M.K.); emilia.kielczynska@student.umw.edu.pl (E.K.); klaudia.kukla@student.umw.edu.pl (K.K.); gabriela.osmulska@student.umw.edu.pl (G.O.); 2 Clinical Department of Allergology and Internal Medicine, Wroclaw Medical University, 50-556 Wroclaw, Poland

**Keywords:** SIBO, atopy, allergy, microbiota, asthma, urticaria

## Abstract

Small intestinal bacterial overgrowth (SIBO) is an increasingly recognized condition that influences immune responses. It may be linked to atopic disorders such as bronchial asthma (BA), food allergies (FA), chronic spontaneous urticaria (CSU), and mast cell activation syndrome (MCAS). The aim of our study was to perform a structured literature search to assess the possible correlation between SIBO and the presentation of atopic disorders. The prevalence of SIBO was highest in patients with BA (60–100%) and FA (50–87.5%), followed by MCAS (30.9%) and CSU (27.9%). The diagnosis of SIBO was based on lactulose or glucose breath tests. SIBO exacerbated symptoms of atopic diseases, and treating it within BA and MCAS improved the symptoms, in contrast to CSU. The present evidence suggests a possible crosslink between SIBO and atopic manifestations. Bacterial overgrowth appears to trigger the Th2 immune response via the mucosal pathway and low-grade endotoxemia. These result in the increased synthesis of interleukins involved in allergic reactions (IL-4, IL-5, IL-13). Further studies are essential to confirm the clinical significance of this association. The “gut–allergy axis” may offer new therapeutic options and possibly improve quality of life in patients with atopy.

## 1. Introduction

**Figure 1:**
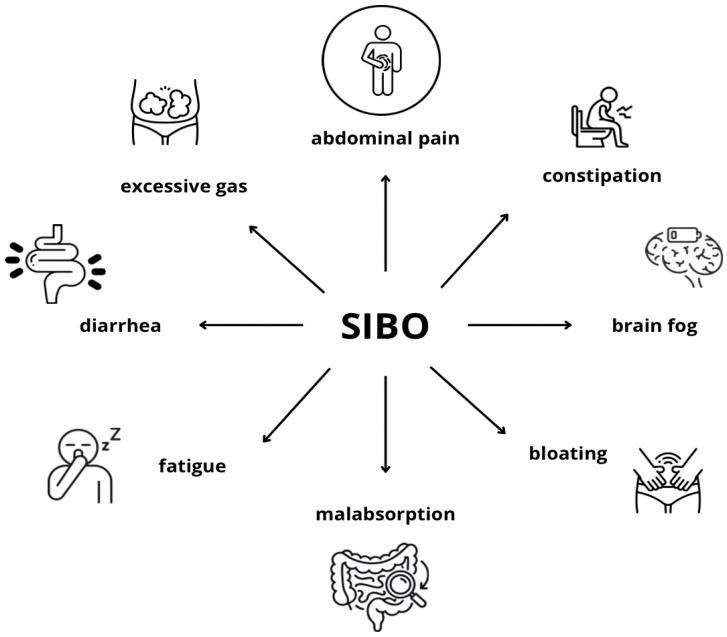
Symptoms associated with SIBO.

Small intestinal bacterial overgrowth (SIBO) is a dysbiotic condition characterized by an increased bacterial population in the small intestine, equal to or greater than 10^5^ CFU/mL. It is primarily associated with impaired gastrointestinal motility, leading to the prolonged retention of food in the gut, and with increased levels of bacteria from the *Enterobacteriaceae* family, mainly *Escherichia*, *Klebsiella*, and *Proteus*, often due to repeated antibiotic therapy and poor diet quality [1,2]. SIBO can also be caused by proton pump inhibitors, which raise the gastric pH and reduce the effectiveness of the stomach’s antibacterial barrier [3]. Common symptoms include abdominal bloating, gas, distension, and diarrhea [4] [Figure 1]. Diagnosis can be non-invasive, using breath tests with lactulose or glucose, or invasive, via small intestinal biopsy cultures (mainly from the distal duodenum), which are considered the gold standard [5]. As its prevalence increases worldwide, SIBO is becoming an increasingly significant clinical concern [2]. This review aims to evaluate the role of SIBO in the development and presentation of symptoms in atopic disorders.

### 1.1. Effects of SIBO on Inflammatory and Immune Responses

The impact of SIBO on inflammatory and immune responses is complex. The overgrowth of bacterial colonies increases luminal lipopolysaccharide (LPS) levels, leading to local mucosal inflammation [1]. Microbial antigens activate epithelial Toll-like receptor 4 (TLR4), triggering the release of epithelial alarmins IL-33 and IL-25 and thymic stromal lymphopoietin (TSLP). These cytokines activate group-2 innate lymphoid cells (ILC2s), which have specific receptors for IL-33 (T1/ST2), IL-25 (IL-17RB), and TSLP (TSLPR) [6]. Once activated, ILC2s initiate type 2 inflammation through two pathways.

### 1.2. Direct Pathway

ILC2s can present antigens to naïve CD4+ T cells via MHC II-TCR interactions, promoting Th2 cell differentiation. During this process, complement component C3 is activated, and its fragment C3a enhances MHC II-dependent T-cell activation while skewing differentiation toward Th2 cells [7]. Costimulatory molecules also contribute: ILC2s express OX40L, which interacts with OX40 on CD4+ T cells to amplify their expansion. IL-4 further induces GATA3 expression, the master transcription factor for Th2 differentiation [8].

### 1.3. Indirect Pathway

ILC2-derived IL-13 activates CD11b+ dendritic cells, which then migrate to draining lymph nodes and promote the differentiation of naïve CD4+ T cells into Th2 cells [6]. Differentiated Th2 cells secrete IL-4, IL-5, IL-9, and IL-13, establishing the typical type 2 cytokine environment linked to atopic disorders. IL-4 promotes immunoglobulin class switching to IgE in B cells, which then binds to FcεRI on mast cells and basophils, triggering their activation and degranulation. IL-4 also enhances Th2 polarization through a positive feedback loop [8]. IL-5 aids in recruiting and maturing eosinophils, while IL-4 and IL-13 stimulate the epithelial production of eotaxins (CCL11 and CCL26), leading to eosinophil accumulation [9]. IL-13 further contributes to smooth muscle hyperreactivity, goblet cell hyperplasia, and mucus overproduction [6]. Local inflammation disrupts epithelial tight junctions [10], increasing intestinal permeability and allowing the translocation of bacterial LPS into systemic circulation. Chronic low-grade endotoxemia can then further boost type 2 inflammation in susceptible individuals through dysregulated TLR4 signaling [11]. Collectively, these mechanisms provide a plausible link: SIBO-induced epithelial activation triggers a type 2 immune response, while systemic low-grade endotoxemia amplifies and sustains Th2 polarization.

### 1.4. Effects of SIBO on Symptoms of Atopic Disorders

**Figure 2:**
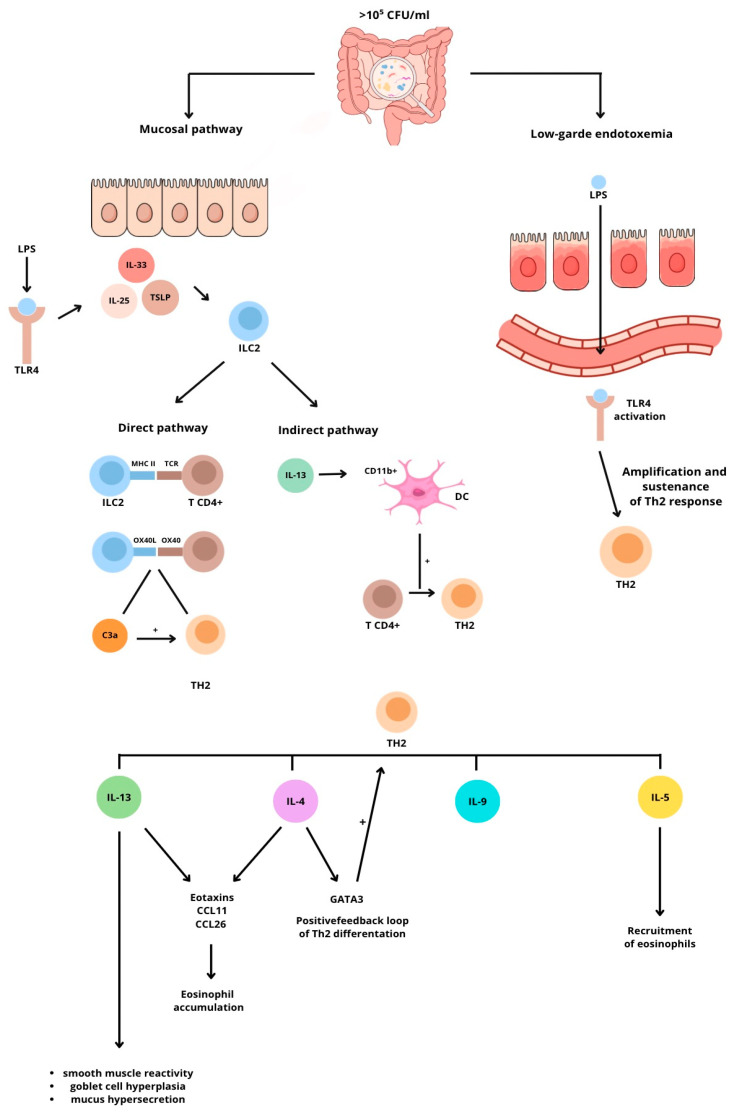
Influence of SIBO on inflammatory and immune responses.

The described molecular pathways can contribute to the worsening of symptoms in allergic diseases such as mast cell activation syndrome (MCAS), bronchial asthma (BA), food allergies (FA), and chronic spontaneous urticaria (CSU) as follows. MCAS—most symptoms of this disorder are linked to hyperreactive mast cells [10,11]. SIBO can increase IL-4 levels, which promotes class switching to IgE in B cells—these immunoglobulins bind to FcεRI on mast cells, triggering degranulation [8]. BA—a disorder primarily associated with hyperreactive bronchi and an inflammatory state [12]. SIBO can lead to increased levels of IL-4, IL-5, and IL-13. This can heighten eosinophil activity (IL-5 by recruiting and maturing eosinophils, and IL-4 and IL-13 by promoting their accumulation through increased eotaxin production), thereby increasing inflammation. IL-13 can further worsen symptoms by causing bronchoconstriction (in bronchial smooth muscles) and increased mucus secretion (goblet cell hyperplasia) [6]. FA—food allergies often involve bloating, chronic abdominal pain, and disrupted gut motility. SIBO, characterized by bacterial overgrowth in the small intestine, may lead to bloating through increased microbial fermentation. Chronic low-grade inflammation can lead to disrupted gut motility (e.g., smooth muscle constriction induced by IL-13) and heightened pain sensitivity [13]. CSU—by increasing IL-4 levels and activating mast cells and basophils, SIBO can raise histamine levels and amplify symptoms like pruritus and edema associated with urticaria [8] (Figure 2).

## 2. Methodology

The literature discussed in this narrative review was identified through searches conducted in PubMed, Embase, Web of Science, and Google Scholar. The structured literature search terms included “small intestinal bacterial overgrowth”, “SIBO”, “allergy”, “atopic dermatitis”, “asthma”, “urticaria”, and “food allergy”, used individually and in combination. Studies were included when they discussed the correlation between SIBO and atopic disorders, had general relevance to the topic and scientific soundness, and were either a case report or original work, presented as a complete, peer-reviewed article. Reviews were considered when they offered essential conceptual or historical context. We imposed neither language nor time restrictions during the search. Animal-based studies were excluded. The following data were sought from each study: population (understood as a group of patients), type of atopy (atopic asthma, food hypersensitivity, chronic spontaneous urticaria, facial erythema, rhinitis, atopic dermatitis, mast cell activation syndrome), SIBO definition and method of diagnosis used, and intervention. Exclusion criteria involved retracted articles, duplicate records across databases, non-peer-reviewed material, conference abstracts, and studies lacking relevance to the main theme of our review. In total, 6 references were included in this work after the selection process.

## 3. Results

### 3.1. Prevalence of SIBO in Atopic Patients

The prevalence of SIBO among atopic patients appears higher than in the general population, but this difference does not always reach statistical significance [14]. Individual diseases vary in terms of co-occurrence with SIBO—in mast cell activation syndrome (MCAS), the prevalence is 30.09%; in chronic spontaneous urticaria (CSU), it is 27% [15]; in bronchial asthma, it is 60–100% [12,16]; and in food allergies, it presents in 50–87.5% of cases [13]. The diagnosis is mostly achieved based on the results of breath tests—lactulose (LBT) and glucose (GBT)—the simplest and cheapest available diagnostic tools for SIBO. One factor contributing to differences in some studies is the high variability in sensitivity and specificity—20–93% for GBT and 30–86% for LBT, according to The North American Consensus [17]. Participants’ age can also affect the results, as children tend to have a higher prevalence of atopic disorders than adults [13]. Furthermore, the application of different diagnostic thresholds (e.g., 15 ppm vs. 20 ppm for hydrogen) across studies likely contributes to the inconsistent prevalence rates of SIBO reported in atopic populations. These diagnostic discrepancies represent a major methodological limitation that necessitates the cautious interpretation of pooled data. In Table 1, we present the different thresholds for SIBO diagnosis used in the studies included in the review.

SIBO can affect the presentation of atopic disorders. A paper by Campanati et al. [15] notes that patients with SIBO reported higher baseline symptom levels, such as pruritus, compared to those with *H. pylori* infection. The effect of treating SIBO varies by disease; for example, it can significantly improve quality of life in patients with bronchial asthma [12,16] and MCAS [18]. However, in conditions like CSU, the impact of such treatment is limited. Key gaps in the current research include the lack of large-sample studies and the heterogeneity among the groups studied. Overall, this review supports the idea that SIBO may influence the clinical presentation of atopy, and, in some cases, its diagnosis and treatment may enhance patients’ quality of life.

### 3.2. SIBO and Mast Cell Activation Syndrome

The prevalence of SIBO in MCAS patients was notably higher than in healthy controls [18], suggesting a potential, important interplay between intestinal immune regulation, motility disturbances, and microbial homeostasis.

**Figure 3:**
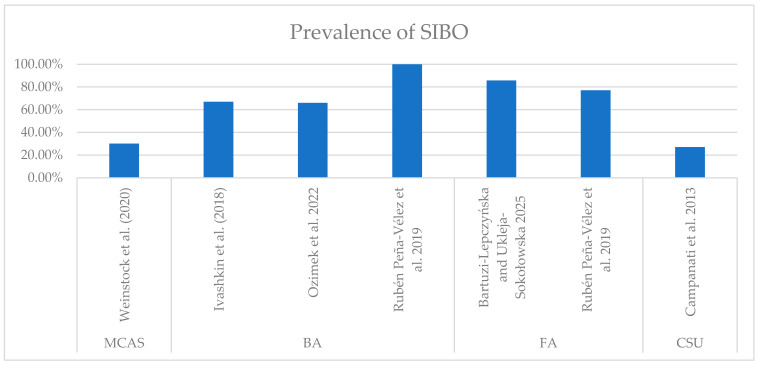
Prevalence of SIBO in atopic disorders (FA—food allergies, BA—bronchial asthma, MCAS—mast cell activation syndrome, CSU—chronic spontaneous urticaria) [12,13,14,15,16,18].

In the study by Weinstock et al. [18], a positive SIBO diagnosis was defined as an increase of ≥20 ppm in the hydrogen concentration from baseline within the first 90 min of the lactulose breath test. A diagnosis of MCAS was established if patients exhibited typical symptoms of mast cell activation involving at least two organ systems, along with at least one of the following criteria: elevated mast cell mediators, clinical improvement with mast-cell-targeted therapy, or increased intestinal mast cell density. A total of 139 MCAS subjects (116 females, 23 males; mean age 46.6 ± 16.9 years) and 30 healthy controls from a prior study (19 females, 11 males; mean age 44 ± 14.0 years) were included. In 66.2% of MCAS patients, gastrointestinal (GI) symptoms (Figure 3) appeared before other MCAS-related manifestations. Moreover, the Rome IV criteria for IBS were met in these MCAS patients, with 39.6% meeting the IBS mixed, 22.3% the IBS constipation, and 18.7% the IBS diarrhea criteria.

**Figure 4:**
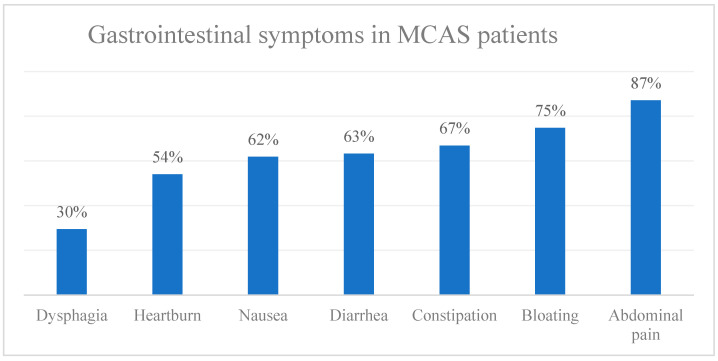
Gastrointestinal symptoms in MCAS patients.

The authors observed that methane excretion in a plateau pattern was common among MCAS patients (10.1% had levels ≥ 10 ppm and 24.5% had levels 3–9 ppm). However, higher methane levels were more frequently associated with IBS constipation than with hydrogen-positive SIBO (42.9% vs. 9.3%, *p* = 0.02). A total of 74 patients received antibiotic therapy to alleviate GI symptoms—67.6% had a marked improvement, 5.4% had a partial improvement, and 21.6% had no improvement. Due to adverse events, antibiotic therapy was discontinued in 9.5% of patients. Antibiotic therapy targeting bacterial overgrowth resulted in marked or partial improvements in most patients, highlighting SIBO as a potentially modifiable factor in the management of GI symptoms in MCAS (Figure 4).

### 3.3. SIBO and Bronchial Asthma

Our literature search identified three studies examining the link between SIBO and bronchial asthma. All studies diagnosed SIBO using the lactulose hydrogen breath test. A positive result was defined as an increase of 15 ppm above baseline by Ivashkin et al. [16] or 20 ppm by Peña-Vélez et al. [12]. Ozimek et al. [12] were the only authors to also analyze the fecal short-chain fatty acid (SCFA) profile. Across the studies, the prevalence of SIBO in patients with bronchial asthma was high. Ivashkin et al. reported SIBO in 67% of asthmatic patients. A similar result was seen in Ozimek et al.’s study, with SIBO diagnosed in 66% of participants.

This topic was further explored in another article that focused on a pediatric population. The highest prevalence was observed in the Peña-Vélez et al. study [13], where all asthmatic patients (100%) were diagnosed with SIBO. Asthma was the third-most-reported atopic disorder among SIBO patients in this study. It should be emphasized that, in this study, not only people with asthma were tested for SIBO; patients with other atopic conditions, such as cow’s milk protein allergy (CMPA), food allergies, allergic rhinitis, urticaria, and atopic dermatitis, were also included. This may have affected the measurement of prevalence in the asthmatic group. Therapeutic interventions targeting SIBO included rifaximin only, probiotics only, or rifaximin + probiotics. We summarize the treatment strategies in Table 2. In both clinical trials, patients received asthma pharmacotherapy—long-acting beta-2-adrenergic agonists and inhaled glucocorticoids.

Before therapy, elevated IgE levels were observed in patients with SIBO. A significant reduction in IgE levels was observed after rifaximin therapy. Primarily, IgE decreased in the group receiving rifaximin + probiotic. Moreover, spirometric parameter FEV1 was improved in both the rifaximin-only and rifaximin + probiotic groups, indicating an improved respiratory status [16].

The authors observed a 2–3-fold lower hospitalization rate after SIBO treatment compared with before treatment. During the 12-month follow-up, 63% of SIBO-positive patients required one hospitalization. Patients treated with rifaximin only were hospitalized more frequently; they accounted for 79% of hospitalizations, and 21% were for those receiving antibiotics plus probiotics. Moreover, 37% of SIBO-positive patients required no hospitalization, and all were in the rifaximin + probiotic group [16].

In Ozimek et al.’s study [12], the authors compared the fecal SCFA profile among patients receiving therapy. Their main findings were the following:

- A significant reduction in isoacids and the isoacid/acid ratio in the group receiving rifaximin + probiotic compared with both the control group and the rifaximin-only group;
- A similar decrease in the isoacid/acid ratio among patients using probiotics only;
- An increase in overall SCFA content, especially acetic and butyric acids, observed in the probiotic-only group. The authors interpreted this as an improvement in the pre-epithelial and epithelial regions of the intestines, meaning that increased SCFA levels indicate improved mucous secretion and pH normalization, reduced permeability, and better epithelial regeneration. Normalization of anaerobic conditions occurred in all groups. There were no changes in fecal SCFA content; the authors suspected that one month was too short a time to expect such a change.

### 3.4. SIBO and Food Allergies (FA)

The available evidence suggests that the prevalence of SIBO may be higher in patients with food allergies compared to those without such conditions. This association indicates a potential link between immune dysregulation and alterations in the small-bowel microbiota.

In the cross-sectional study by Bartuzi-Lepczyńska and Ukleja-Sokołowska [14], an investigation was conducted among patients with various suspected food hypersensitivities based on the skin prick test (SPT) and clinical presentation. Patients were diagnosed with SIBO using a 10 g lactulose load and breath measurements taken every 20 min, in accordance with the North American Consensus on breath testing. Positive results were found in 85.7% of patients with a history of food allergy and in 78.3% of patients without confirmed allergies. However, the authors emphasize that this difference was not statistically significant. Based on these findings, the authors conclude that SIBO may have a higher prevalence among individuals with food allergies. This may indicate the need for further research to determine whether the observed difference is indeed not statistically significant. The authors also suggest that Western dietary patterns may contribute to the dysbiosis characteristic of SIBO. Additionally, they note that the growing prevalence of food allergies may be associated with broader civilizational changes. It is also important to highlight the study’s limitations, including the small sample size and the considerable heterogeneity of the general population. Regardless of the observed outcomes, the high detection rate of SIBO in patients with gastrointestinal complaints, with or without atopy, deserves particular attention.

This topic is further explored in another article that focuses on a pediatric population. In Peña-Vélez et al.’s study [13], 70 patients with chronic abdominal pain (CAP) were enrolled (41 females and 29 males), aged 2 to 18 years, representing a pediatric population. The children were evaluated for allergic diseases such as allergic rhinitis, asthma, and urticaria, all of which were confirmed by the SPT. In cases of food allergy, specific IgE levels were assessed, while atopic dermatitis was diagnosed based on clinical presentation. The study did not provide information about distinct diagnostic criteria for cow’s milk protein allergy (CMPA). All participants underwent SIBO assessment using the lactulose breath test (LBT). Each patient received lactulose at a dose of 0.5 g/kg, with a maximum dose of 10 g. Breath samples were collected every 20 min over three hours. A test result was considered positive if the hydrogen levels exceeded the baseline values by 20 parts per million (ppm). The analysis revealed that the number of patients with SIBO and those without the condition was equal, with 35 individuals in each group. The authors conclude that certain types of allergies, such as CMPA, allergic rhinitis, and asthma, are associated with SIBO. Furthermore, they observed that patients with both SIBO and allergic diseases were younger than SIBO-positive patients without allergic manifestations. A similar pattern was noted across specific age groups.

### 3.5. SIBO and Chronic Spontaneous Urticaria (CSU)

The role of SIBO in the clinical presentation of chronic spontaneous urticaria (CSU) was assessed by Campanati et al. [15], who studied 51 participants (17 men and 43 women). Patients with CSU were diagnosed with SIBO (by glucose breath test, with H_2_/CH_4_ production > 12 ppm above basal values) or Helicobacter pylori infection (by urea breath test). The prevalence of SIBO was higher than in the general asymptomatic population—i.e., 27.1% (*n* = 13; in the general population, 0–12.5%), with that of *H. pylori* infection at 22.9% (*n* = 11)—similar to that of the general populations of industrialized countries. Patients positive for both were excluded. Treatment included rifaximin 1200 mg/day for one week for SIBO and combined antibiotic therapy as suggested by the American College of Gastroenterology Guidelines. Patients were assessed using the urticaria activity score (UAS) and dermatology life quality index (DLQI) before and 1 month after treatment. The results showed significantly higher baseline scores for UAS and worse DLQI scores for SIBO—these patients had more severe symptoms, but the eradication of *H. pylori* had a greater impact on patients’ quality of life. HP patients reported nearly 50% lower UAS and DLQI scores, whereas SIBO patients reported only 10% lower scores. The authors emphasize that H. pylori eradication was achieved with broad-spectrum antibiotic therapy and that this therapy could have eradicated other subclinical infections related to CSU. The authors conclude that the results, while supporting the eradication of *H. pylori* infection, do not support SIBO treatment in patients with CSU.

## 4. Discussion

The prevalence of small intestinal bacterial overgrowth (SIBO) appears to be higher in atopic patients than in the general population, but this does not reach statistical significance in every study. Patients positive for SIBO in general have more severe symptoms of atopic diseases, with bronchial asthma and food allergies as the most common and CSU/MCAS as the least correlated. Our findings align with the gut–immune axis, which emphasizes the role of the intestinal microbiota in regulating the immune system. There are two main mechanisms that likely explain the influence of SIBO on atopic diseases. The first is the mucosal pathway, which involves mucous cells and TLR4 receptors. These stimulate the secretion of IL-33, IL-25, and TSLP, thereby activating ILC2 cells. This leads to the direct and indirect activation of the Th2 immune response, leading to the increased synthesis of IL-4, IL-5, and IL-13—interleukins typically involved in allergic reactions. The second pathway is characterized by low-grade endotoxemia, resulting from a dysfunctional gut barrier in an inflammatory state. This leads to TLR4 activation throughout the body. Patients with asthma, food allergies, mast cell activation syndrome, and chronic spontaneous urticaria should be more frequently tested for SIBO. A critical assessment of the studies’ quality identified several methodological limitations that may affect the certainty of the conclusions drawn. In the cross-sectional studies conducted by Ivashkin et al. (2018) [16], Ozimek et al. (2022) [12], and Bartuzi-Lepczyńska and Ukleja-Sokołowska (2025) [14], the authors did not justify their sample sizes. This omission could increase the risk of a type II error—where a lack of statistical significance might result from an inadequate sample size rather than the absence of a clinical correlation. Furthermore, there is no information on non-responders, which complicates the assessment of how representative these groups are of the broader population of patients with SIBO. Caution should be exercised when interpreting the results of Campanati et al. (2013) [15]. Although the authors report no statistically significant demographic differences, raw data analysis indicated notable clinical disparities between the SIBO group and the control group—for example, in smoking rates (61.5% vs. 29.2%) and polypragmasia (46.2% vs. 29.2%). The lack of statistical control for these confounders reduces the credibility of this study according to the Newcastle–Ottawa scale. In the study by Peña-Vélez et al. (2019) [13], the cross-sectional design limits the ability to determine the sequence of events. There is also no early microbiota verification or follow-up, which means that the results only demonstrate the co-occurrence of SIBO in certain populations and do not establish a causal or correlational relationship with atopic disorders. The study by Weinstock et al. (2020) [18] has severe evidentiary limitations due to significant gender homogeneity. We observed the over-representation of women (83%) and a lack of control for confounding variables. This suggests that the observed effects are specific to the female population and require verification in more balanced cohorts. The collective analysis of the quality of evidence indicates that future studies on SIBO should focus on a prospective observational design with rigorous control of external factors to avoid selection and interpretation bias. Evidence linking SIBO to atopic manifestations remains modest. One can argue about which comes first—atopy or SIBO. We believe that this relationship can be bidirectional. On one hand, SIBO can cause a leaky gut, which may induce general inflammation, leading to the activation of mast cells and worsening allergic reactions. On the other hand, atopy, by triggering inflammation in the body, can decrease the activity of the migrating motor complex (MMC), leading to bacterial buildup in the intestine. Moreover, patients with atopy often use drugs such as glucocorticosteroids, which can alter the microbiome. More studies are needed to establish a correlation/causation. We propose randomized controlled trials with significantly larger, homogeneous populations, as well as longitudinal studies, to determine when patients, e.g., those with asthma, develop SIBO.

## 5. Conclusions

The present evidence suggests a correlation between SIBO and atopic manifestations, including asthma, food allergies, and mast cell activation-related symptoms. Overgrowth of the bacterial population may lead to the activation of the Th2 response and the translocation of LPS, inducing low-level endotoxemia and the activation of the Th2/TLR-4 pathway. This activates and promotes a Th2 immune response, present in atopic disorders, which is characterized by elevated levels of IL-4, IL-5, and IL-13. However, it has yet to be determined whether this is a causal or a correlational link. There is still limited literature on this topic. Further studies are needed to establish the clinical significance of this association. An understanding of the gut–allergy axis may lead to new therapeutic options and possibly improve quality of life for atopic patients.

## Figures and Tables

**Table 1:** Diagnostic methods for SIBO and diagnostic thresholds in the analyzed studies.

Study (Author, Year)	Atopic Disorder	Population	Substrate	Diagnostic Threshold	Measured Gases (H_2_, CH_4_)	Prevalence of SIBO (%)
Weinstock et al. 2020 [18]	MCAS	139 MCAS subjects (116 F, 23 M, 46.6 ± 16.9 years)	Lactulose	20 ppm	H_2_, CH_4_	30.09%
Ivashkin et al. 2018 [16]	BA	45 patients with BA, 21 males and 24 females	Lactulose	15 ppm	H_2_	67%
Ozimek et al. 2022 [12]	BA	30 patients with acute exacerbation of allergic BA (16 F, 14 M), with an average age of 37.7 ± 10.1 years	Lactulose	Not specified	H_2_	66%
Rubén Peña-Vélez et al. 2019 [13]	BA FA	70 were included, 41 females and 29 females, aged 2–18 years, with CAP	Lactulose	20 ppm	H_2_	100% 77%
Bartuzi-Lepczyńska and Ukleja-Sokołowska 2025 [14]	FA	44 patients aged 21–73 (26 females and 18 males)	Lactulose	20 ppm	H_2_	85.7%
Campanati et al. 2013 [15]	CSU	51 patients—17 males and 34 females	Glucose	12 ppm	H_2_, CH_4_	27%

**Table 2:** Treatment strategies among analyzed studies.

Authors	Number of Patients	SIBO Present + Absent −	Treatment		Probiotic Composition
Ivashkin et al.	15	+	Rifaximin 800 mg/day for 7 days		Not applicable
	15	+	Rifaximin 800 mg/day for 7 days	Probiotic 1 capsule 3×/day for a month	*Bifidobacterium bifidum* not less than 1 × 10^9^ CFU *Bifidobacterium longum* not less than 1 × 10^9^ CFU *Bifidobacterium infants* not less than 1 × 10^9^ CFU *Lactobacillus rhamnosus* not less than 1 × 10^9^ CFU
Ozimek et al.	10	+	Rifaximin 200 mg 3×/day for 7 days		Not applicable
	10	+	Rifaximin 200 mg 3×/day for 7 days	Probiotic 1 capsule 3×/day for a month	3.0 × 10^9^ CFU/capsule with at least: *Lactobacillus gassery KS-13* *Lactobacillus gasser LAC-343* *Lactobacillus ramnosus LCS-742,* *Bifidobacterium bifidum G9-1* *Bifidobacterium longum MM-236* *Bifidobacterium longum MM-236 M* *Bifidobacterium infantis M-63* *Bifidobacterium breve M16V type T* *Bifidobacterium lactis B1-04*
	10	−	Probiotic 1 capsule 3×/day for a month		

## Data Availability

No new data were created or analyzed in this study. Data sharing is not applicable to this article.
